# A Dispensary Clinic.—Cancer of the Rectum

**Published:** 1896-08-29

**Authors:** 


### Medical Progress and Hospital Clinics.

[*The Editor will be glad to receive offers of co-operation and contributions from members of the profession. All letters should be addressed to* The Editor, at the Office, 428, Strand, London, W.C.]

### A DISPENSARY CLINIC.—CANCER OF THE RECTUM.

In answer to the bell there next appeared a tall, pale, tremulous man of seventy, bearing in his face the aspect of having suffered much.

Doctor: Well! what is the matter with you?

Patient: Pain in the back.

D.: Whereabouts?

P. (Putting his hand on the lower part of his sacrum): Down at the bottom of the bone here.

D.: When is it worst?

P.: Its always there, day and night; I can get no rest, and the pain has taken all my appetite. I cannot eat, and am wasting away.

D.: How long have you had it?

P.: About three months, not more, and it gets worse.

D.: How are the bowels?

P.: Bad. I cannot get them properly open; but when I go they move freely.

D.: How do you mean they move freely if you cannot get them open?

P.: I mean that what comes comes free enough, but there is only a little of it.

D.: Do you ever part with a solid motion?

P.: No; not for three months past. It's just a little squashy stuff; but it comes easy and without pain.

D.: The pain then is not made worse by the action of the bowels?

P.: No, it does not seem to make any difference. But I'm always wanting to go. Sometimes I get up twenty times in the night thinking I can do something, but very little comes; I've no power.

D.: Have you any piles?

P.: No. Nothing ever comes down; but twice lately I've parted with a little blood—only a little though.

D.: Is there any mattery discharge at the seat?

P.: No; the seat is all right. There is no pain there; it's in the bone at the bottom of my back.

D.: Have you any trouble with your water?

P.: No. I pass it a little more often than I used to, but there is no pain in passing it.

D. to Student: Now, what is there which will cause a pain like this to come on in an elderly man? You notice that he does not sit down gingerly and on one side, as if he had external piles; he flops down exhausted, without taking any special care how he sits. This same point of his mode of moving and sitting down goes against other conditions which sometimes cause considerable pain in these parts. There are various affections of the coccyx, and the muscles attached to it, which cause very wearing pains, but they are peculiarly apt to be aggravated by certain movements, and patients so affected generally sit down very carefully. Then fissure of the anus will in some cases give rise to pain, which may be constant, but it certainly will be worse after defecation. Internal piles will sometimes cause peculiarly wearing and distressing pains, but then they usually give rise to pretty free hæmorrhage. Enlarged prostate, especially when complicated with abscess, will cause intense pain in the back; but there will be other symptoms connected with the urinary passages, and the patient will not sit down freely. There is one condition which is worth noting which may give rise to much pain, to small, ineffectual evacuations leaving a desire to pass more, and ultimately to much ill-health, and that is the impaction of a concretion or a mass of fæces in the rectum. But such cases are different from this one. Here we have no bearing down. He often thinks he could get his bowels moved, and so he tries again, but he is not driven to it by tenesmus. Again, in cases of impaction, as also of internal piles, the pain is mostly relieved by lying down, whereas here the pain is as bad at night as in the day. I know nothing that will produce the condition from which this man suffers except a growth, not necessarily in the bowel, but pressing upon it. I have seen much the same symptoms produced by a sarcoma growing from the anterior surface of the sacrum. Let us examine and see what there is to be found.

[On examination the anus was seen to be dry and free from all remains of scars and from all traces of old piles. On introducing the finger the rectum was noted to be ballooned. It contained no fæces and in the lower part no growth was felt. Higher up, however, the roof of the pelvis was one hard mass of infiltration, nodular, of great hardness, with small outlying shotty masses between the main growth and the mucous membrane, but nowhere was any ulceration to be felt. The growth extended forwards and was felt to surround the upper portion of the rectum, so that not even the tip of the finger could be introduced into the upper continuation of the bowel. The whole growth was absolutely immovable.]

D. to Student examining: Now, first of all you notice that the external sphincter is somewhat tight, which is probably a reflex condition, for there is nothing about the anus to account for it. Next you perceive that the finger enters an empty space so that the point of the finger can be freely moved about without touching anything. That is the so-called ballooning of the rectum. It occurs in many conditions and it would not do to attribute too much importance to it. No doubt its essential cause is spasm of the anus preventing the escape of flatus, especially when obstruction above prevents its return. Next applying your finger to the posterior surface of the rectum, well up against the coccyx, and tracing it upwards, you find that after going up about two inches your finger becomes separated from the hollow of the sacrum by a hard nodular mass, and passing the finger forwards you notice that the whole roof of the rectum, so to say, almost up to the bladder in front, is filled in by this growth, about the middle of which you can with some difficulty find the entry of the bowel coming down from above, into which however you cannot introduce your finger. Now you must note first of all the extent of the growth; next its hardness, there is no "giving" as if there might be an abscess up above; then it is absolutely fixed; and lastly, you must notice that the mucous membrane lies smoothly over it without any ulceration. Notice also that at certain points, lying apparently between the mucous membrane and the growth, there are little nodules over which the mucous membrane can be made to slide. Now withdraw your finger. Do it gently and slowly, for wherever there is spasm of the anus withdrawal is apt to cause pain. Now, lastly, you notice that you have not caused any bleeding by what you have done. It is a case of malignant disease infiltrating the tissues of the bowel, but, as yet, not producing ulceration as far as is within reach of the finger.

Now what can we do for this poor man? He is suffering principally from pain, from broken sleep, and from want of appetite and consequent extreme debility. (On getting off the table he was very faint, but was immediately revived by a few drops of sal volatile.) But if you tried to feed him he would soon suffer from obstruction, and if you relieved his pain by opiates you would probably arrive at the same result. In my opinion the best course will be to do colotomy. It is obviously impossible to remove the growth, but colotomy would probably relieve him of the constant disturbance at night; it would enable him to take food without danger of obstruction; it would enable us to give him opium without fear; and it might possibly in some degree modify the progress of the disease. I do not believe that a colotomy will check the growth of a carcinoma, but when we have to do with a growth like this, the mass of which at any rate has not begun to ulcerate, it is, I think, fair to believe that a colotomy efficiently done, with the production of such a spur as to keep the lower bowel empty, is the best means we possess of preventing those necrotic processes which are the cause of a large proportion of the troubles of cancer of the rectum. We will then send him into hospital. You must remember, however, that this is by no means a typical case of cancer of the rectum. From its commencement carcinoma is usually attached much more closely to the mucous membrane, and much sooner leads to ulceration and to projection into the bowel than is the case here, as you saw a few weeks ago in a case here, in which, with complete ulceration and with the projection of fungating masses into the lumen of the bowel, the growth was limited, there being a clean stretch of untouched mucous membrane upon the posterior surface of the bowel, and the finger being able to reach healthy tissue above, so that, advanced as the disease was in the part affected, it might have been within the reach of surgery but for its unfortunate position, attached to and probably invading the bladder at its base. Here we have a very different condition, a widespread scirrhous infiltration which cannot be removed.

